# Ligand-based chemoinformatic discovery of a novel small molecule inhibitor targeting CDC25 dual specificity phosphatases and displaying *in vitro* efficacy against melanoma cells

**DOI:** 10.18632/oncotarget.5473

**Published:** 2015-10-13

**Authors:** Alessandra Capasso, Carmen Cerchia, Carmen Di Giovanni, Giuseppina Granato, Francesco Albano, Simona Romano, Emmanuele De Vendittis, Maria Rosaria Ruocco, Antonio Lavecchia

**Affiliations:** ^1^ Department of Molecular Medicine and Medical Biotechnology, University of Naples Federico II, 80131 Naples, Italy; ^2^ Department of Pharmacy, “Drug Discovery” Laboratory, University of Naples Federico II, 80131 Naples, Italy

**Keywords:** cancer, CDC25 phosphatases, drug discovery, cell cycle, melanoma cells

## Abstract

CDC25 phosphatases are important regulators of the cell cycle and represent promising targets for anticancer drug discovery. We recently identified NSC 119915 as a new quinonoid CDC25 inhibitor with potent anticancer activity. In order to discover more active analogs of NSC 119915, we performed a range of ligand-based chemoinformatic methods against the full ZINC drug-like subset and the NCI lead-like set. Nine compounds (3, 5–9, 21, 24, and 25) were identified with *K*_i_ values for CDC25A, -B and -C ranging from 0.01 to 4.4 μM. One of these analogs, 7, showed a high antiproliferative effect on human melanoma cell lines, A2058 and SAN. Compound 7 arrested melanoma cells in G2/M, causing a reduction of the protein levels of CDC25A and, more consistently, of CDC25C. Furthermore, an intrinsic apoptotic pathway was induced, which was mediated by ROS, because it was reverted in the presence of antioxidant N-acetyl-cysteine (NAC). Finally, 7 decreased the protein levels of phosphorylated Akt and increased those of p53, thus contributing to the regulation of chemosensitivity through the control of downstream Akt pathways in melanoma cells. Taken together, our data emphasize that CDC25 could be considered as a possible oncotarget in melanoma cells and that compound 7 is a small molecule CDC25 inhibitor that merits to be further evaluated as a chemotherapeutic agent for melanoma, likely in combination with other therapeutic compounds.

## INTRODUCTION

Cell division cycle 25 proteins (CDC25s) are dual-specificity phosphatases (DSPs), acting as key regulators of the cell cycle. Indeed, CDC25 controls the activity of cyclin-dependent kinases (CDKs), by removing inhibitory phosphates from tyrosine and threonine residues on the phosphate binding loop [1–2]. Regulation of protein levels and activity of CDC25 facilitates the orderly progression through the cell cycle; furthermore, these enzymes play an important role as checkpoint regulators for handling DNA damage caused by UV light, ionizing irradiation, or chemicals. Therefore, the misregulation of CDC25s could be pivotal for causing genomic instability. In humans, CDC25 belongs to a multigene family consisting of three forms: CDC25A, -B, and -C [3]. CDC25A plays an extensive role in assisting both G1/S and G2/M progression, by dephosphorylating CDK4-Cyclin D [4] and CDK6-Cyclin D complexes [5], as well as CDK1-Cyclin B, CDK2-Cyclin A and CDK2-Cyclin E complexes [6–7]. CDC25B is responsible for the initial activation of CDK1-Cyclin B complex at the centrosome during the G2/M transition, which is then followed by a complete activation of CDK1-Cyclin B complexes by CDC25C in the nucleus at the onset of mitosis [8]. CDC25B is also able to dephosphorylate and activate CDK2-Cyclin A and CDK2-Cyclin E complexes [9–10]. CDC25C is present in each cell cycle phase and regulates the G2/M transition, by targeting CDK1-Cyclin B complex [11–12]. However, evidence was presented that all CDC25 forms can regulate both G1/S and G2/M transitions [13]. In order to assure a controlled progression through each cell cycle phase and thus maintain the genomic integrity, a tight regulation of CDC25 phosphatases activity is needed, both in unperturbed cell cycle and in response to DNA damage checkpoints. This regulation depends upon post-translational mechanisms such as phosphorylations, sub-cellular relocalization and proteasome-mediated degradation [14], together with p53-dependent transcriptional repression of the three CDC25 phosphatases [15–16]. Misregulation of CDC25s has been shown to cause unscheduled entry into mitosis, spontaneous mutagenesis and sensitisation to DNA damaging agents [17–18]. Further roles have been proposed for these phosphatases in diverse areas, such as centrosome amplification [19] and steroid receptor coactivation [20]. Overexpression of CDC25A, CDC25B or both was reported in a wide variety of human malignancies including breast, thyroid, laryngeal, esophageal, gastric, hepatocellular, ovarian, endometrial, prostate, and colorectal, non-Hodgkin lymphomas as well as in gliomas, neuroblastoma and melanoma [8] and was commonly associated with both tumor aggressiveness and poor prognosis [21]. With regard to CDC25C, only a few studies showed an overexpression of this form in cancers [22–23]. However, growing evidence suggests that the overexpression of CDC25C could be underrated because of the non-consideration of its alternative splicing [21, 24]. In the complex, all these observations indicate that CDC25s are promising targets for the development of anti-cancer drugs.

**Figure 1:**
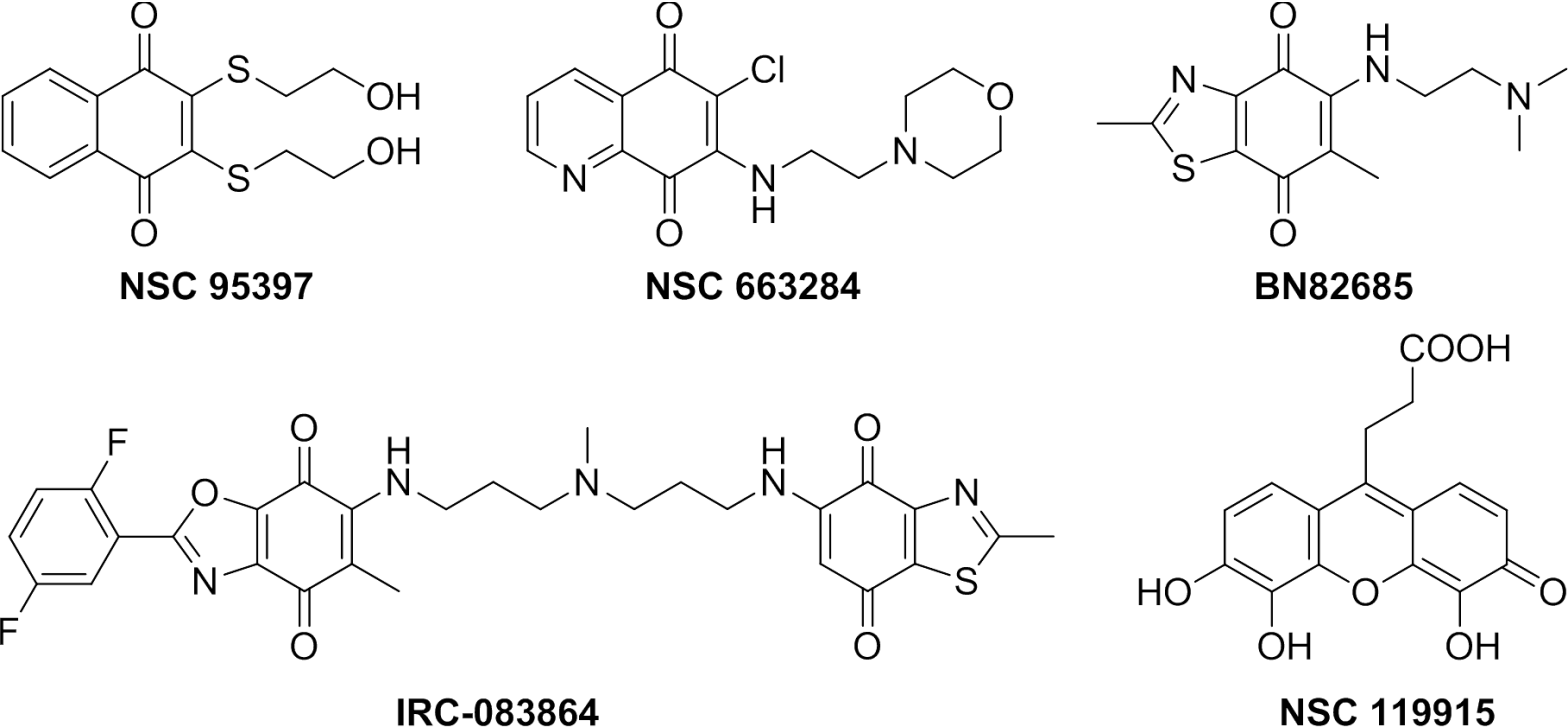
Known quinone-containing CDC25 Inhibitors

Over the past few years, several synthetic and natural molecules with different structural features targeting CDC25 activity have been reported. Reviews by Lavecchia *et al*. provide a comprehensive overview of the current discovery of CDC25 inhibitors [25–27]. Most of the known CDC25 inhibitors belong to various chemical classes including phosphate bioisosteres, electrophilic entities, and quinone-based structures. It is thought that there are three possible mechanisms through which these molecules inhibit CDC25s and other phosphatases, i.e. reversible inhibition through binding to the active site of CDC25s [28–29], irreversible inhibition of CDC25s through a direct binding with the inhibitor [30–31], or oxidation of the critical cysteine residue in the catalytic domain (CX_5_R) by reactive oxygen species (ROS) generated in cultured cells treated with quinone derivatives [32–33]. This latter mechanism could be consistent with the non-selective inhibition of CDC25 phosphatases by quinone-type inhibitors. Moreover, ROS may oxidize other phosphatases, as well as unrelated cysteine-based enzymes, and therefore quinone-containing agents could potentially trigger several unrelated events in cells. To date, many of the most potent CDC25 inhibitors are quinone-containing compounds, which inhibit all three forms of CDC25 in an unselective manner. Among these, NSC 95397 [28], NSC 663284 [30], BN82685 [34] and IRC-083864 [35] are representative potent inhibitors (Figure 1).

We have previously reported the discovery of a new quinonoid CDC25 inhibitor (NSC 119915 in Figure 1) by means of a structure-based high-throughput virtual screening [36]. This compound displayed irreversible inhibition kinetics with *in vitro K*_i_ values for CDC25A and -B of 0.07 and 0.08 μM, respectively; furthermore, NSC 119915 generated an increase of the intracellular ROS level, arrested cells in the G0/G1 and G2/M phases of the cell cycle, and significantly inhibited the growth of human MCF-7 breast, PC-3 prostate, and K562 leukaemia cancer cell lines.

It is known that melanoma, one of the most aggressive tumors, is very refractory to any conventional therapies. Therefore, a great effort has been devoted to discover new molecules helpful in the treatment of this cancer acting through the modulation of key pathways of cell proliferation. CDK2 and CDK6, cyclins D1, E, and D3 and phosphatase CDC25A are consistently overexpressed in metastatic melanomas compared with nevus tissue [37–38]. Hence, the potent inhibition displayed by the compound NSC 119915 towards CDC25 could be suggestive of its possible activity even in melanoma, and therefore it was judged as an appropriate choice for studying its activity against melanoma cells.

In this study, we described the use of chemoinformatic and virtual screening (VS) approaches against the full ZINC drug-like subset [39–40] and the NCI (National Cancer Institute) lead-like set to potentially identify more active analogs of our lead compound NSC 119915 and to expand our understanding of structure-activity relationships (SARs). Our strategy, based on atom connectivity similarity and substructure searches, led to the identification of twenty-five analogs of NSC 119915. Among them, nine compounds, that share a same 6-xanthone chemical motif (3, 5–9, 21, 24, and 25), showed an *in vitro* inhibitory activity towards CDC25A, -B and -C, comparable with that exerted by NSC 119915. The effect of these compounds was also evaluated in a cellular context, using the melanoma cell lines A2058 and SAN. The data showed that compound 7 was by far the most effective one in the inhibition of cell proliferation, as emerging from the cytotoxicity tests. Furthermore, compound 7 affected the cell cycle progression, modulated the CDC25 protein levels and triggered the cell death, by inducing an apoptotic program, as evaluated through different markers. In addition, 7 produced an alteration of the cellular redox state and caused a mitochondrial dysfunction, likely associated to a modulation of the Akt pathway.

## RESULTS

### Compound selection using chemoinformatics

**Figure 2:**
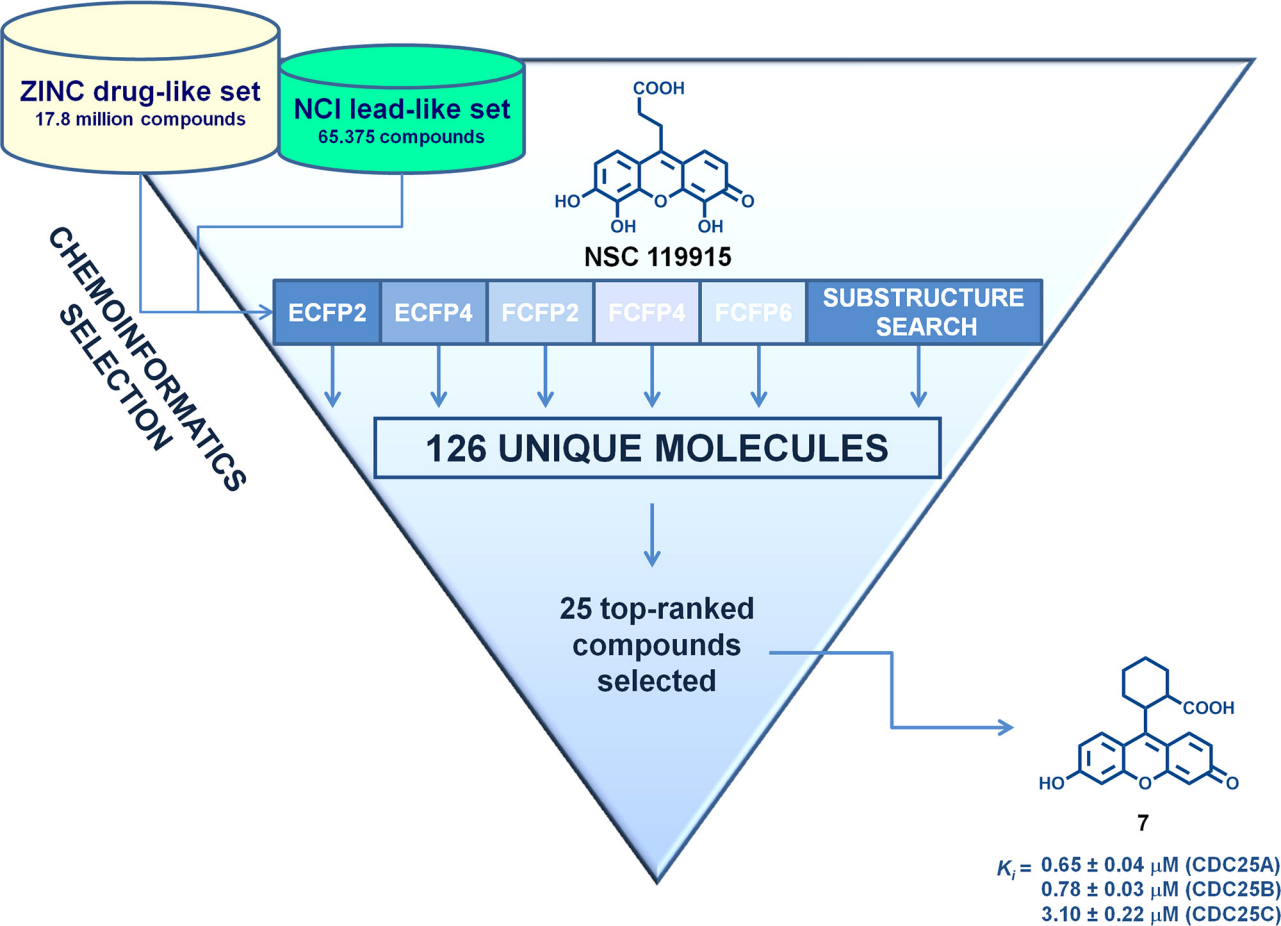
Flow chart of the multiple ligand-based chemoinformatic strategy implemented in this work

As the primary goal of this work was to identify novel structural analogs with increased CDC25 inhibitory potency of lead compound NSC 119915, we applied different chemoinformatic approaches [41–42] against both the ZINC drug-like library and the NCI lead-like set. The general workflow of the multiple ligand-based chemoinformatic approaches implemented in this work is presented in Figure 2.

The first five VS approaches employed molecular fingerprints, which are binary vectors encoding the presence, or absence, of substructural fragments within the molecule and have been successful in recognizing similar molecules in large databases [43]. We employed ECFP2, ECFP4, FCFP2, FCFP4, and FCFP6 to identify close active analogs to our lead NSC 119915, using the Tanimoto coefficient as similarity measure. To enhance the probability of finding 50% of all possible actives, we used the threshold values suggested by Muchmore *et al*. [44] So, a Tanimoto threshold of 0.52 for ECFP2 allowed the selection of 13 compounds, whereas a Tanimoto threshold of 0.43 for ECFP4 gave 12 compounds. For FCFP2, a Tanimoto threshold of 0.75 provided 16 compounds; for FCFP4, a Tanimoto threshold of 0.60 gave 5 compounds; and for FCFP6, a Tanimoto coefficient of 0.45 highlighted 8 compounds. The sixth method utilized was that of the substructure search using the core of lead NSC 119915. By definition, a substructure search identifies molecules that contain a defined molecular fragment, that is, a certain substructure. Of course, such a search will not lead to new scaffolds, but will allow the finding of close analogs and possible variations in the decoration of known molecule classes. The substructure search identified 137 scaffold isosteres of our lead core structure.

The results of the six VS techniques were combined, and a significant number of identical compounds were found; this overall strategy led to a final total of 126 unique compounds, that were predicted by one or more methods to be similar in some way to our active compound. As we were not able to screen this number of compounds by *in vitro* assays, we selected the top-ranked 25 compounds that were purchased or requested from the NCI Developmental Therapeutics Program (DTP) (Table 1). Our decision to select compounds from the top-ranked compounds was to ensure testing of any highly similar (and therefore likely to be active) compounds.

**Table 1:** Compounds identified by multiple ligand-based chemoinformatic protocol 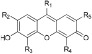

Cpd	Code	R_1_	R_2_	R_3_	R_4_	R_5_	Cpd	Code	R_1_	R_2_	R_3_	R_4_	R_5_
1	NSC158113	CH_3_	H	H	H	H	14	NSC4202	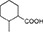	H	I	I	H
2	ZINC 04015433	CH_3_	OH	H	H	OH	15	NSC4905	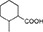	I	I	I	I
3	NSC158115	COOH	H	H	H	H	16	ZINC 04409973	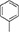	H	Cl	Cl	H
4	NSC158112	CH_2_CH_2_COOH	H	H	H	H	17	ZINC 04352921	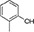	H	Br	Br	H
5	NSC119894	CH=CHCOOH	H	H	H	H	18	NSC2087	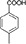	Br	Br	Br	Br
6	NSC119911	CH=CHCOOH	H	OH	OH	H	19	ZINC04261930	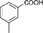	NO_2_	Br	Br	NO_2_
7	NSC119892	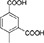	H	H	H	H	20	ZINC03861600	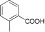	H	OH	OH	H
8	NSC119910	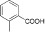	H	OH	OH	H	21	NSC119893	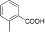	H	OH	OH	H
9	ZINC 03860685	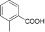	OH	H	H	OH	22	ZINC04822213	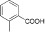	OH	H	H	OH
10	ZINC 13597410	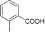	OH	H	H	OH	23	ZINC04582279	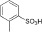	H	H	H	H
11	ZINC05030632	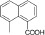	H	H	H	H	24	NSC 119912	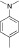	H	OH	OH	H
12	NSC 119888	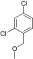	H	H	H	H	25	NSC 119916	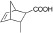	H	OH	OH	H
13	ZINC 05030658	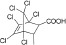	H	H	H	H							

### Effect of the close analogs of NSC 119915 on the phosphatase activity of purified recombinant forms of CDC25

A preliminary screening of the inhibition properties of the close analogs of NSC 119915 was carried out by a fluorimetric assay, that measured the residual phosphatase activity of a recombinant form of CDC25B in the presence of the selected compounds. The solutions of NSC 119915 and of its structural analogs were carefully monitored to avoid artifacts due to precipitation or agglomeration of the compounds. Among the twenty-five structures identified from the multiple ligand-based chemoinformatic approach, eight compounds (2, 10, 12–14, and 16–18) were excluded from the analysis, because endowed with a strong fluorescent signal, which interfered with the emission wavelength of the synthetic substrate 3-O-methylfluorescein phosphate (OMFP) used in the fluorimetric assay. The inhibition of the phosphatase activity of CDC25B by the remaining seventeen analogs was evaluated in the presence of two different concentrations of these compounds (Table 2). The data indicated that compounds 5–9, 21, 24, and 25, that contain a 6-xanthone chemical motif, exerted a concentration-dependent inhibition of the CDC25B phosphatase activity, with a percentage of inhibition comparable to that exhibited by compound NSC 119915 (Supplementary Figure S1). In contrast, compounds 1, 3, 4, 11, 15, 19, 20, 22, and 23 caused a measurable inhibition of the phosphatase activity, only when added at the highest concentration. For this reason, only one of these latter compounds, i.e. 3, was included in the following analysis.

**Table 2:** Residual phosphatase activity of CDC25B in the presence of compound NSC 119915 or its 6-xanthone analogs

Cpd	CDC25B residual activity (%) in the presence of [inhibitor]	
	0.2 μM	1 μM
1	76	9
3	85	62
4	90	28
5	12	2
6	14	2
7	44	1
8	10	1
9	9	0
11	89	44
15	79	18
19	86	70
20	91	24
21	13	1
22	80	0
23	80	14
24	22	3
25	24	1
**NSC 119915**	38	2

**Figure 3:**
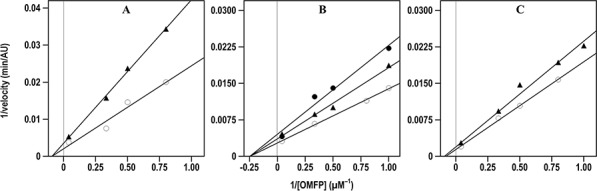
Effect of compound 7 on the Lineweaver-Burk plots of the A. CDC25A B. CDC25B and C. CDC25C phosphatase activity The phosphatase activity was measured through the rate of OMFP hydrolysis as described in the Materials and Methods. The activity was determined either in the absence (empty circles in **A., B.** and **C.**) or in the presence of the following concentrations of compound 7: 0.5 μM (filled triangles in **A.**), 0.25 μM or 0.5 μM (filled triangles or filled circles, respectively, in **B.**), 1 μM (filled triangles in **C.**).

The inhibition properties of the effective inhibitors were better investigated through the kinetic measurement of their *K*_i_ values. For instance, Figure 3 shows the kinetic evaluation of the inhibitory effect of compound 7 on the three CDC25 forms, as representative of the inhibition mechanism exerted by these compounds; indeed, a similar behaviour was observed with the other 6-xanthone derivatives, thus allowing the measurement of the corresponding *K*_i_ values.

As shown in Table 3, the low *K*_i_ values confirmed that these compounds possessed an effective inhibition towards CDC25A, -B or -C. Indeed, the measured *K*_i_ values obtained for the 6-xanthone derivatives were similar to that determined for NSC 119915 and all comprised in relatively small intervals; in particular, the *K*_i_ towards CDC25A ranged between 0.01 and 0.80 μM, and the corresponding intervals for CDC25B and CDC25C were 0.12–2.4 μM and 0.30–4.4 μM, respectively. Furthermore, when considering the inhibition potency of each compound towards the different CDC25 phosphatases, a similar efficacy was observed, because no great differences emerged from the comparison of the respective *K*_i_ values. However, among the three forms, CDC25A showed a slightly higher sensitivity towards the inhibitors, whereas CDC25C had a moderately lower responsiveness. Concerning the mechanism of inhibition, the kinetic measurements showed that the tested compounds had a behaviour similar to that already reported for NSC 119915 [36]. In particular, in the presence of the inhibitors the *K*_M_ value for the substrate OMFP was not essentially modified, whereas the *V*_max_ of phosphatase activity was significantly reduced. Therefore, the tested analogs of NSC 119915 were noncompetitive inhibitors of CDC25 and probably acted in an irreversible manner.

**Table 3:** *K*_i_ values of compound NSC 119915 or its 6-xanthone analogs towards CDC25-A, -B and -C phosphatases

Cpd	*K*_i_ (μM)		
	CDC25A	CDC25B	CDC25C
3	0.28 ± 0.09	2.4 ± 0.39	4.4 ± 0.62
5	0.38 ± 0.12	0.12 ± 0.05	0.39 ± 0.15
6	0.10 ± 0.03	1.1 ± 0.37	1.0 ± 0.41
7	0.65 ± 0.04	0.78 ± 0.03	3.1 ± 0.22
8	0.17 ± 0.07	0.19 ± 0.08	0.30 ± 0.12
9	0.80 ± 0.31	0.44 ± 0.2	1.5 ± 0.48
21	0.14 ± 0.06	0.14 ± 0.05	0.63 ± 0.14
24	0.01 ± 0.005	0.3 ± 0.08	0.40 ± 0.11
25	0.40 ± 0.15	1.1 ± 0.4	0.96 ± 0.38
**NSC 119915**	0.34 ± 0.12	0.10 ± 0.04	0.24 ± 0.13

### Evaluation of CDC25 inhibitors on cell growth rate of A2058 and SAN melanoma cells

**Figure 4:**
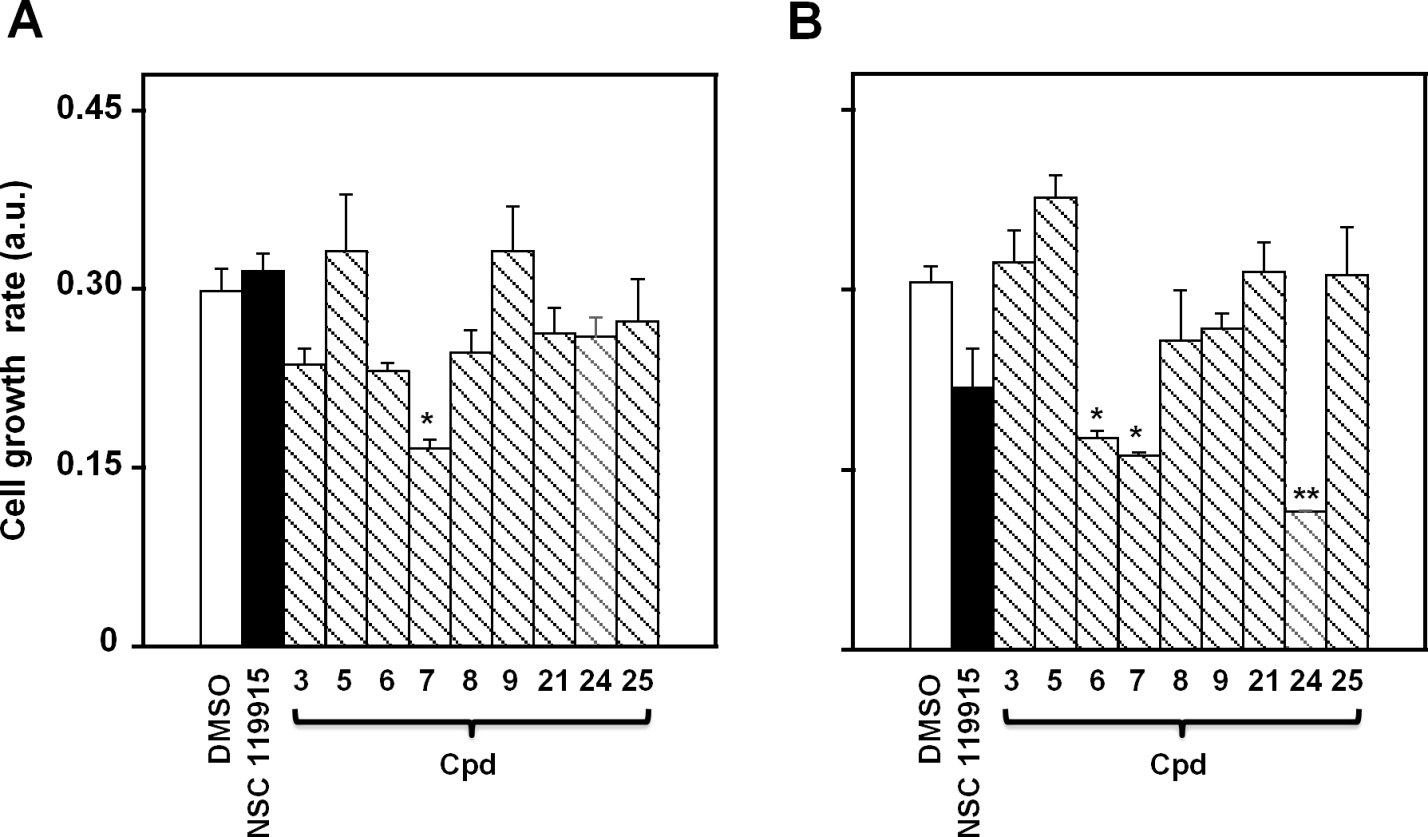
Effect of NSC 119915 and its close analogs on cell growth rate of melanoma cells **A.** A2058 and **B.** SAN cells were incubated for 48 h with 0.5% DMSO as a vehicle control or 100 μM of each of the indicated compounds. The cell growth rate is reported as arbitrary units (a.u.). Data from four independent experiments are reported as the means ± SE. *p < 0.05 and **p < 0.01 compared to control cells.

In a previous work we found a strong antiproliferative action of NSC 119915 on some cancer cell lines [36]. Here, we investigated the effects of NSC 119915 and its selected 6-xanthone analogs on the growth rate of two melanoma cell lines, A2058 and SAN. In particular, the effect of the inhibitors was evaluated after different times of treatment with 25, 50 or 100 μM NSC 119915 or its analogs 3, 5–9, 21, and 24–25. The minimum concentration of inhibitor that caused an evident cytotoxic activity was 100 μM, as demonstrated for cpd 7 in Supplementary Figure S2; therefore, this concentration was almost thoroughly used in the following experiments. Figure 4 shows the cell growth rate of A2058 and SAN cells after 48-h treatment in the presence of 100 μM of the different compounds. As shown in Figure 4A, only compound 7 caused a significant reduction of the cell growth rate of A2058, whereas the other derivatives, as well as NSC 119915, were quite ineffective or caused a not significant reduction of cell growth rate. A similar behaviour was observed after 72-h incubation; indeed, only compound 7 provoked the significant reduction of cell growth rate (data not shown). Figure 4B reports the effect of the inhibitors in SAN cells; in this case, three compounds, i.e. 6, 7 and 24, caused a significant reduction of cell growth rate after 48-h treatment. When the treatment was prolonged up to 72 h, also compound NSC 119915 exerted a significant reduction of cell growth rate (not shown). In conclusion, compound 7 could be representative of the effect of this group of molecules in melanoma cells, because of the significant cytotoxicity observed in both cell lines.

### Effect of compound 7 on cell cycle progression and apoptosis

**Figure 5:**
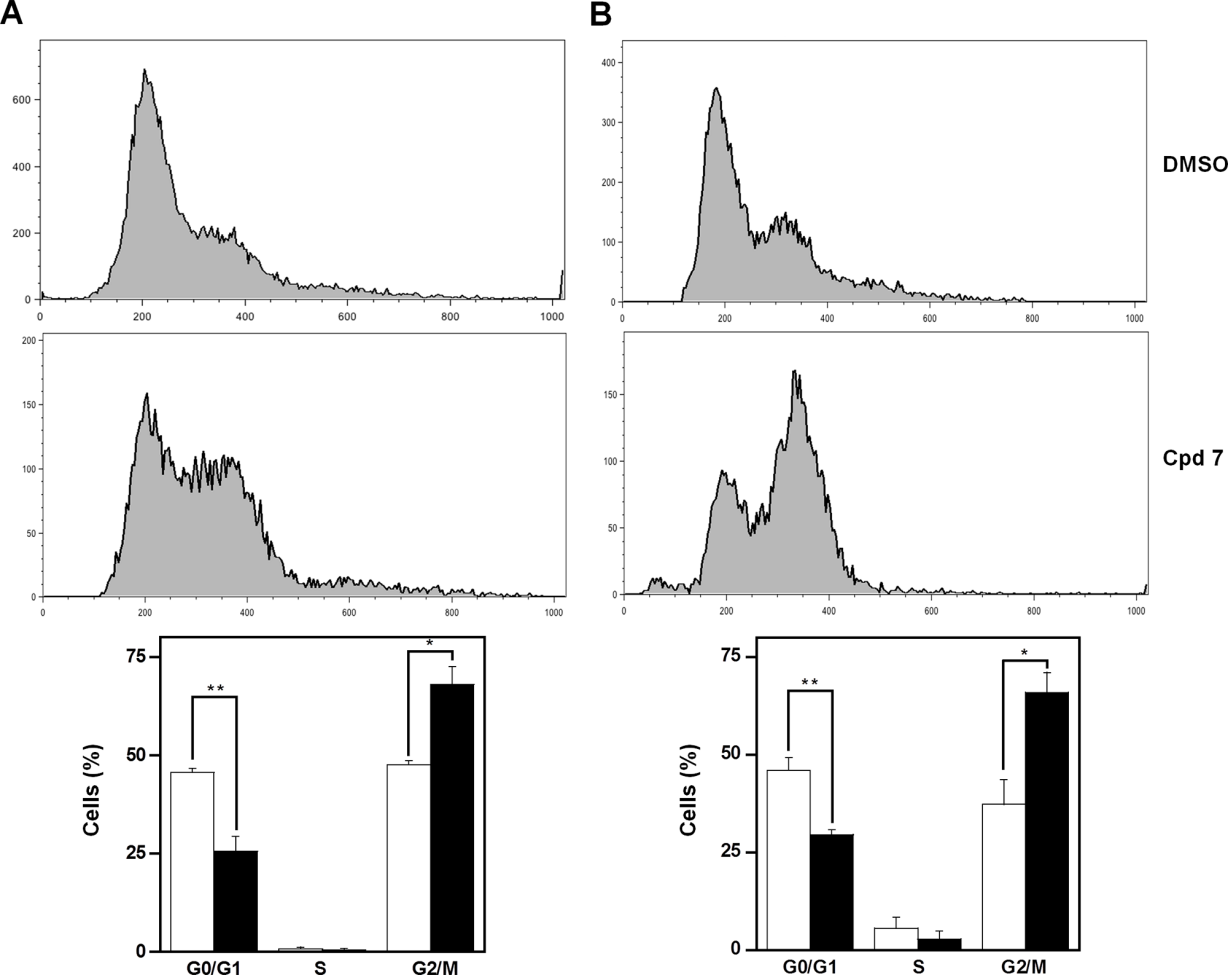
Effect of compound 7 on the distribution of cell cycle phases of A2058 cells The determination of cells in the different phases was evaluated after **A.** 16 h or **B.** 24 h from treatment with 0.5% DMSO or 100 μM compound 7, as described in the Materials and Methods. Histograms, which show the cell percentage among the various phases, were obtained from triplicate experiments and reported as the means ± SE. *p < 0.05 and **p < 0.01 compared to control cells. Vehicle alone, open bars; compound 7, black bars.

As CDC25 phosphatases are key cell cycle regulators, the effect of 7 on cell cycle progression was investigated in detail. To this aim, asynchronously growing A2058 and SAN cells were treated at different times with 100 μM compound 7, and then cell cycle analysis was cytofluorimetrically monitored after propidium iodide (PI) incorporation. Figure 5 shows the time-dependent distribution of the cell cycle in its different phases of A2058 cells. After 16-h incubation with vehicle alone, cells were mainly and almost equally distributed in G0/G1 and G2/M phases (the ratio between them being 0.96), whereas the cellular population in the S phase was essentially undetectable. On the other hand, after 16-h treatment with 7, a significant reduction of cells in G0/G1 phase was evident, accompanied by a significant improvement of the G2/M cell arrest (Figure 5A); in particular, the ratio between G0/G1 and G2/M decreased to 0.38 (*p* < 0.05). A similar behaviour was observed if the incubation was prolonged up to 24 h (Figure 5B); in this case the ratio between G0/G1 and G2/M decreased from 1.23 (untreated cells) to 0.45 (treated cells; *p* < 0.01). A similar general picture emerged from the effect of compound 7 on cell cycle progression of SAN cells (Supplementary Figure S3). In spite of some differences in the relative cell phase distribution, also in these melanoma cells compound 7 caused an increase of cell distribution in the G2/M phase after 16- and 24-h treatment.

**Figure 6:**
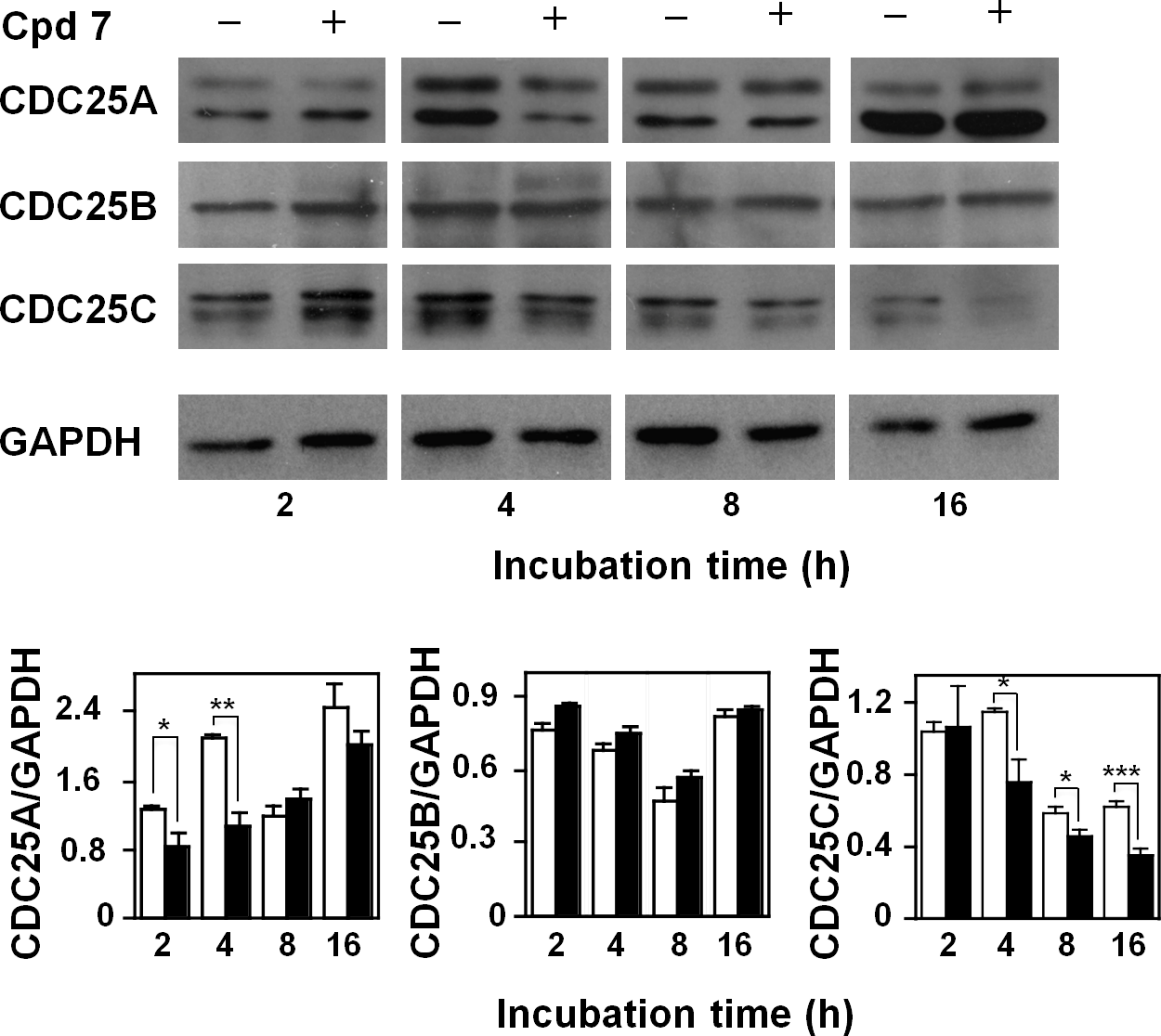
Effect of compound 7 on CDC25A, B and C protein levels Total protein extracts from A2058 cells, incubated with 0.5% DMSO (open bars) or 100 μM compound 7 (black bars) for 2, 4, 8 or 16 h, were analyzed by Western blotting. GAPDH was used as loading control. The doublets observed in the immunoblots detecting CDC25A or CDC25C were due to the common presence of multiple isoforms of these proteins. Densitometric analysis is shown in the lower panel. Data from triplicate experiments were reported as the means ± SE. *p < 0.05, **p < 0.01, ***p < 0.001, compared to control cells. Other details as described in the Materials and Methods.

It is known that CDC25 phosphatases are crucial regulators of cell cycle progression, and the previous *in vitro* results indicated that compound 7 was a potent inhibitor of CDC25. To better investigate the effect of this inhibitor at the molecular level, we evaluated if the treatment of A2058 cells with 7 affected also the protein levels of the three CDC25 forms. The Western blotting analysis reported in Figure 6 shows that compound 7 provoked an early reduction of the CDC25A protein levels up to 4 h compared to untreated cells; this reduction disappeared under prolonged incubation times. The treatment of melanoma cells with compound 7 also caused a reduction of the CDC25C protein levels, although with a late kinetics. In particular, a progressive and significant reduction of CDC25C protein levels was observed, starting from 4 h and continuing up to 16 h. On the other hand, the protein levels of CDC25B were unaffected by treatment of A2058 cells with 7 up to 16 h. An earlier decrease of CDC25C protein levels was observed even in SAN cells treated with compound 7 (Supplementary Figure S4). These overall results suggest that the modulation of the CDC25 protein levels induced by 7, in particular of the -A and more evidently of the -C form, could be related to the arrest of melanoma cells in G2/M observed in the presence of this inhibitor.

**Figure 7:**
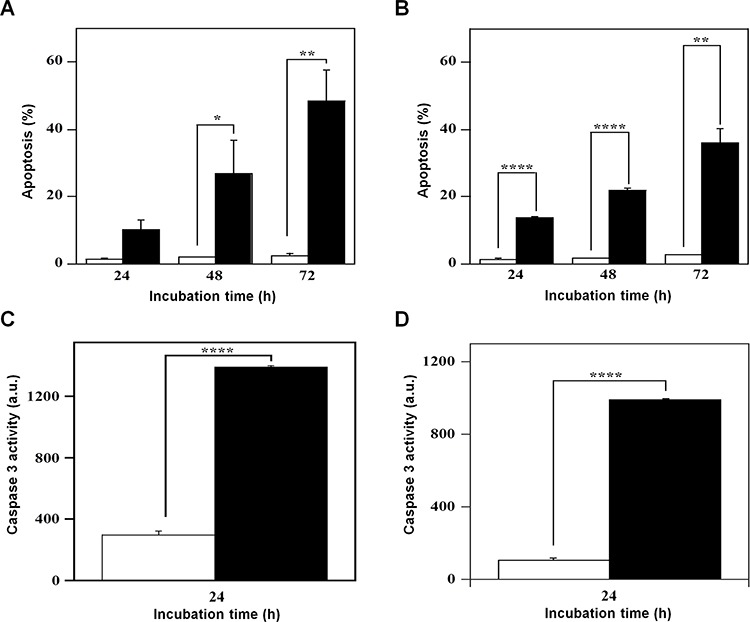
Effect of compound 7 on the apoptosis of A2058 and SAN cells The apoptotic process was evaluated in A2058 (panels **A.** and **C.**) and SAN (panels **B.** and **D.**) cells either through the determination of the number of cells with a subdiploid DNA content (**A.** and **B.**) or by measuring the caspase-3 enzymatic activity (**C.** and **D.**). Cells were treated with 0.5% DMSO (open bars) or 100 μM compound 7 (black bars) for the indicated incubation times. Apoptosis was expressed as a percentage, whereas caspase-3 activity was reported as arbitrary units (a.u.). Data from triplicate experiments were reported as the means ± SE. *p < 0.05, **p < 0.01, ****p < 0.0001, compared to control cells. Other details as described in the Materials and Methods.

The effect of 7 on cell growth and cycle progression could suggest the beginning of a cell death program, a hypothesis investigated through various methodological approaches. PI incorporation followed by flow cytometric analysis was used to detect the effect of 7 on the number of nuclei with a sub-diploid content, a typical hallmark of apoptosis. A time-dependent increase of apoptosis was evident in both A2058 and SAN cells and in particular, the effect of 7 on cell death program was already evident after 24-h treatment (Figure 7). In A2058 cells the increase of apoptosis became significant at 48 h, and continued at least up to 72-h incubation (Figure 7A). In SAN cells a similar behaviour was observed and the increase of apoptosis was significant even at 24-h treatment (Figure 7B). To further investigate on the capacity of 7 to induce apoptosis, the enzymatic activity of caspase-3, the final effector of apoptotic program, was monitored. Indeed, this inhibitor provoked a significant increase of the enzymatic activity of caspase-3 in both A2058 (Figure 7C) and SAN cells (Figure 7D).

**Figure 8:**
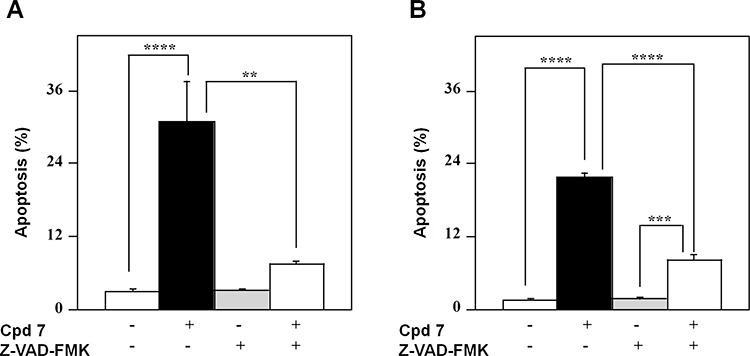
Effect of Z-VAD-FMK, a pan-caspase inhibitor, on the apoptotic process induced in melanoma cells by compound 7 A2058 (panel **A.**) and SAN (panel **B.**) cells were treated with 0.5% DMSO or 100 μM compound 7 and incubated in the absence or in the presence of 100 μM Z-VAD-FMK; the apoptosis was evaluated after a 48-h incubation through the determination of the number of cells with a subdiploid DNA content. Data from triplicate experiments were reported as the means ± SE. **p < 0.01, ***p < 0.001 and ****p < 0.0001 compared to respective control cells. Other details as described in Materials and Methods.

To confirm that the pro-apoptotic effect of 7 was caspase-mediated, the 48-h treatment of A2058 and SAN cells with compound 7 was also carried out in the presence of an irreversible pan-caspase inhibitor, such as Z-VAD-FMK. The level of apoptosis caused by 7 was significantly decreased in the presence of this inhibitor in both A2058 (Figure 8A) and SAN cells (Figure 8B), thus demonstrating that the pro-apoptotic effect of 7 was mainly caspase-dependent. Taken together, all these data support the hypothesis that cell cycle arrest in G2/M phase caused by 7 could evolve in an apoptotic process.

### Effect of compound 7 on ROS generation and mitochondrial membrane potential

**Figure 9:**
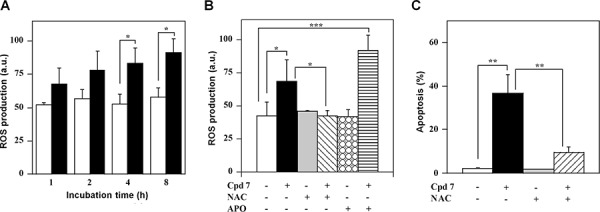
ROS production and their involvement in the apoptotic process of A2058 cells, as induced by treatment with compound 7 **A.** Time-dependent measurement of ROS production. Cells were incubated with 0.5% DMSO (open bars) or 100 μM compound 7 (black bars) and then the intracellular ROS level was measured. **B.** Effect of antioxidant molecules on ROS production. The ROS level was also measured in cells untreated or pretreated with NAC or apocynin after a 4-h incubation with DMSO or 7. **C.** Effect of NAC on apoptosis. The PI incorporation was evaluated in cells untreated or pretreated with NAC after a 48-h incubation with DMSO or 7. ROS production was expressed as a.u., and apoptosis as a percentage. Data from triplicate experiments were reported as the means ± SE. *p < 0.05 and **p < 0.01 compared to control cells. Other details as described in the Materials and Methods.

The high redox reactivity of quinonoid molecules, like compound 7, could greatly perturb the intracellular redox state. Indeed, a time-dependent increase of the ROS level was observed upon treatment of A2058 cells with 7 and this enhancement became significant after 4-h (Figure 9A). A similar behaviour was observed also in SAN cells (Supplementary Figure S5A). The oxidant effect of 7 was also evaluated after cell pre-treatment with two antioxidant molecules, such as N-acetyl-cysteine (NAC) and the specific inhibitor of NADPH oxidase, apocynin. To this aim, A2058 and SAN cells were pre-incubated for 1 h with 10 mM NAC or 45 min with 0.5 mM apocynin and then treated for 4 h with 100 μM compound 7. The increase of ROS level was prevented by the NAC treatment in both A2058 (Figure 9B) and SAN cells (Supplementary Figure S5B). In contrast, apocynin did not prevent the increase of ROS level caused by 7 in both melanoma cell lines, even though this antioxidant raised the basal intracellular redox state of untreated SAN cells. The cytotoxic activity of 7 could be mediated by the observed increase of ROS level. Under this concern, pre-incubation of A2058 and SAN cells with an anti-oxidant molecule could prevent the apoptotic process; indeed, the apoptosis was in a great part reverted by the cellular pre-treatment with NAC (Figure 9C and Supplementary Figure S5C). Taken together, all these findings suggest that 7 altered the intracellular redox state of melanoma cells, thus mediating the observed cytotoxicity.

**Figure 10:**
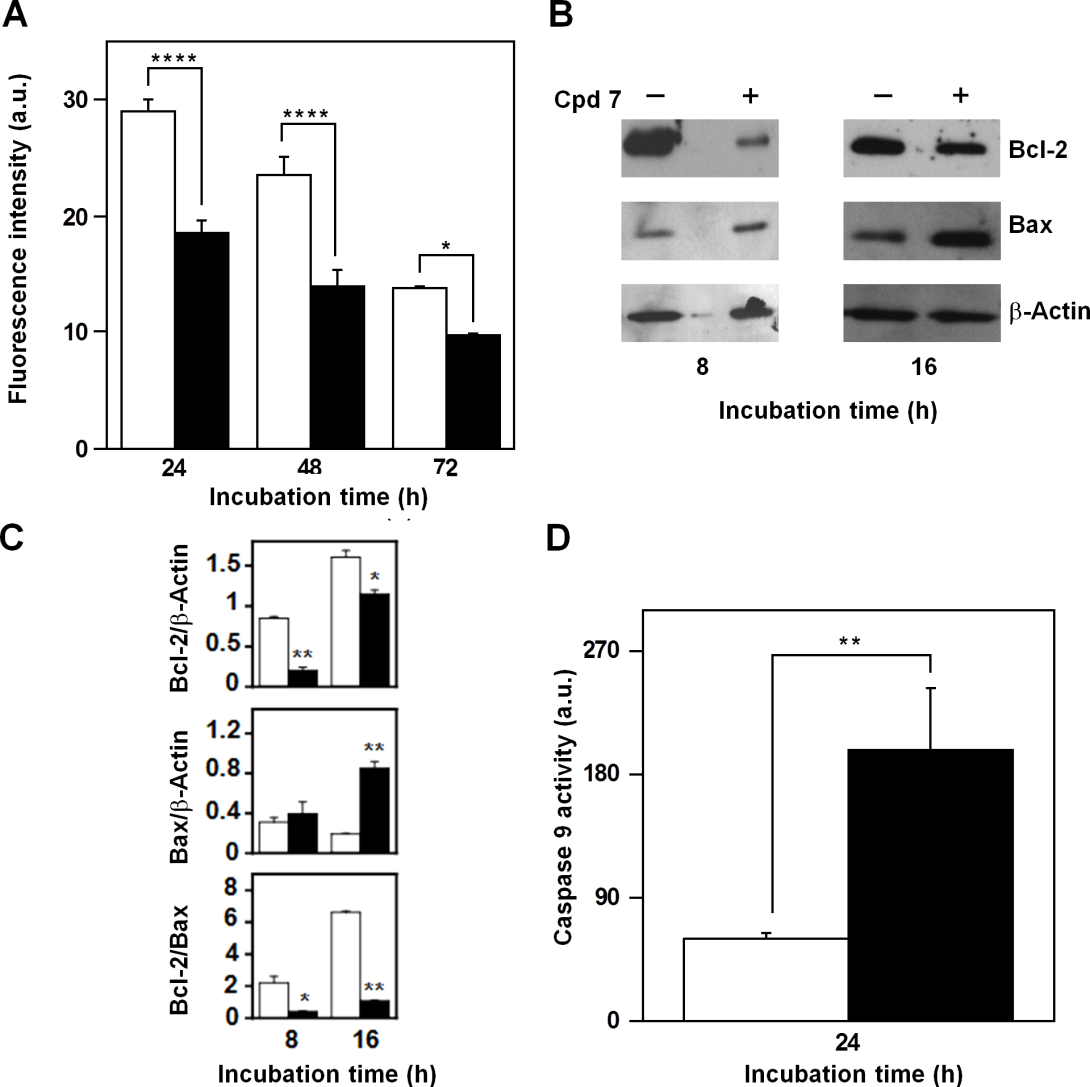
Effect of compound 7 on some apoptotic mitochondrial markers in A2058 cells **A.** Measurement of the mitochondrial membrane depolarization. Cells were treated for the indicated times with 0.5% DMSO (open bars) or 100 μM compound 7 (black bars). **B.** Evaluation of Bcl-2 and Bax protein levels. Total protein extracts from A2058 cells, incubated with 0.5% DMSO or 100 μM compound 7 for 8 or 16 h, were analyzed by Western blotting. β-actin was used as loading control. **C.** Densitometric analysis of the Bcl-2 and Bax protein levels, as well as of the Bcl-2/Bax ratio. **D.** Determination of the caspase-9 enzymatic activity. Total protein extracts from A2058 cells, incubated with 0.5% DMSO (open bars) or 100 μM compound 7 (black bars) for 24 h, were assayed for caspase-9 activity. Data from triplicate experiments were reported as the means ± SE. *p < 0.05, **p < 0.01, ****p < 0.0001, compared to control cells. Other details as described in the Materials and Methods.

Mitochondria represent the primary source of ROS, as well the target of ROS action, and therefore, compound 7 could affect their functionality in melanoma cells. A decrease of the fluorescent signal, corresponding to a reduction of the mitochondrial membrane potential, was already evident after 24-h treatment of A2058 cells with 7 and then continued at least up to 72 h (Figure 10A). Proteins belonging to the B-cell lymphoma-2 (Bcl-2) family are involved in the modulation of the mitochondrial functionality [45–46]. Therefore, we have evaluated the levels of the anti-apoptotic protein Bcl-2, as well as that of the pro-apoptotic Bcl-2-associated X protein (Bax), after incubation with 7 (Figure 10B). In A2058 cells the incubation with the inhibitor caused a clear reduction of the Bcl-2 level after 8-h treatment, and this decrease was still evident after 16 h; on the other hand, a clear increase of Bax was observed after 16-h incubation with 7 (Figure 10C). The densitometric analysis also included the measurement of the Bcl-2/Bax ratio, and its significant reduction observed after 8 and 16 h represents a better tool to reveal the regulation of the apoptotic process. To further investigate the occurrence of a mitochondrial-mediated apoptosis induced by 7, the caspase-9 activity was assayed. A clear increase of caspase-9 activity was measured in A2058 cells after 24-h incubation with 7 (Figure 10D). The effect of compound 7 was investigated also in SAN cells and the overall results were similar to those observed in A2058 (Supplementary Figure S6). In particular, in SAN cells both levels of Bcl-2 and Bax were reduced by the inhibitor treatment; however, the most crucial parameter for monitoring the pro-apoptotic effect of 7, the Bcl-2/Bax ratio, significantly decreased in the presence of 7, because of the greater reduction of Bcl-2 compared to that of Bax. All these data indicate that the increase of ROS levels associated to an alteration of typical mitochondrial markers could contribute or mediate the cytotoxic effect exerted by 7 in melanoma cells.

### Effect of compound 7 on Akt activation and p53 protein levels

**Figure 11:**
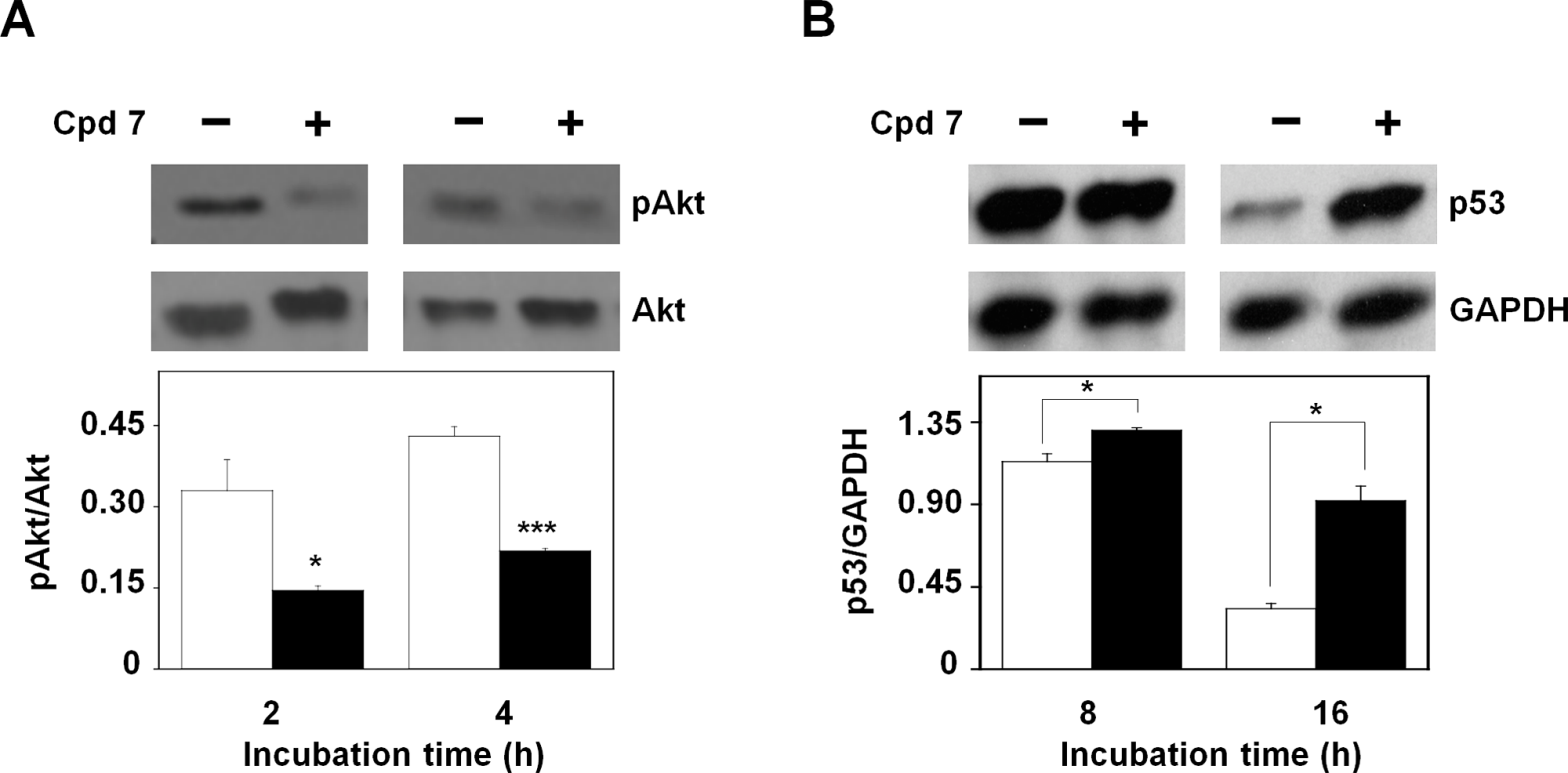
Effect of compound 7 on pAkt and p53 protein levels **A.** Evaluation of pAkt protein levels. Total protein extracts from A2058 cells, incubated with 0.5% DMSO or 100 μM compound 7 for 2 or 4 h, were analyzed by Western blotting using an antibody raised against pAkt (Ser473). Akt was used as loading control. Densitometric analysis shown in the lower panel. **B.** Evaluation of p53 protein levels. Total protein extracts from A2058 cells, incubated with 0.5% DMSO or 100 μM compound 7 for 8 or 16 h, were analyzed by Western blotting. GAPDH was used as loading control. Densitometric analysis shown in the lower panel. Data from triplicate experiments were reported as the means ± SE. *p < 0.05, ***p < 0.001, compared to control cells. Other details as described in the Materials and Methods.

The molecular mechanisms that regulate the cytotoxic potential of 7 in melanoma cells were further investigated through the evaluation of the activation state of protein kinase B (pAkt), one of the key proteins involved in the control and regulation of cell survival [47–48]. To this aim, the protein levels of pAkt have been analyzed after 2- and 4-h incubation of A2058 cells with 7. As shown in Figure 11A, the inhibitor caused an early reduction of the protein level of pAkt with respect to total Akt; indeed, this decrease was already evident after 2-h and remained detectable until 4-h incubation. An early reduction of Akt activation was observed also in SAN cells (Supplementary Figure S7). It is known that Akt regulates the process of cell survival by phosphorylating different substrates, directly or indirectly involved in the apoptotic program [49]. One of these targets is p53, a protein with a tumor-suppressor activity that regulates the cell cycle, as well as the expression of several genes involved in the apoptosis [49]. In particular, Akt negatively regulates the apoptosis, by enhancing the degradation of p53 via its phosphorylation, as well as by promoting the nuclear localization and binding of this factor to human murine double-minute 2 (MDM2) protein, a negative regulator of p53 [49]; therefore, we tested if the CDC25 inhibitor 7 affected the protein levels of p53. As shown in Figure 11B, the higher level of p53 compared to control, already evident after 8-h incubation of A2058 cells with compound 7, became significant after 16-h treatment, because of the reduction of its basal level in untreated cells. Also in SAN cells a time-dependent reduction of the basal level of p53 was observed, together with a higher level of this protein measured in treated cells after 16-h incubation (not shown). We can suggest that the modulation of p53 protein levels represents one of the molecular events linked to the early decrease of pAkt caused by 7.

## DISCUSSION

By combining experimental and computational methods, we have identified a set of inhibitors of CDC25 phosphatases, which are key elements in the control of the cell cycle in eukaryote cells, in normal conditions as well as in response to cell damage, and whose overexpression is associated to a wide variety of cancers [8, 21]. Specifically, we employed a number of ligand-based chemoinformatic methods in the rational selection of 25 close analogs of our lead NSC 119915 [36], that were predicted to possess inhibitory activity against the CDC25A, -B and -C phosphatases. Interestingly, among the 25 candidates, nine compounds sharing the same 6-xanthone motif (3, 5–9, 21, 24, and 25) caused a dose-dependent inhibition of the CDC25B phosphatase activity. Kinetic analyses revealed that these compounds inhibited the three different CDC25 proteins in a noncompetitive manner with *K*_i_ values comparable to those of the lead compound NSC 119915. However, among the three forms, CDC25A showed a slightly higher sensitivity towards the inhibitors, whereas CDC25C had a moderately lower responsiveness.

As CDC25 phosphatases, in combination with other cell cycle regulators, have been proved to be of determinant significance for melanoma growth and/or transformation [37–38], we evaluated the effects of NSC 119915 and its close analogs on the growth rate of two melanoma cell lines, A2058 and SAN. Compound 7 [2-(6-hydroxy-3-oxo-3*H*-xanthen-9-yl)cyclohexanecarboxylic acid] resulted as the only common inhibitor of the cell growth rate of both A2058 and SAN melanoma cells. However, in SAN cells, also the analogs 6 [(*E*)-3-(4,5,6-trihydroxy-3-oxo-3*H*-xanthen-9-yl)acrylic acid] and 24 [(1*R*,3*R*,4*S*)-3-(4,5,6-trihydroxy-3-oxo-3*H*-xanthen-9-yl)bicyclo[2.2.1]hept-5-ene-2-carboxylic acid], as well as in some experimental conditions also NSC 119915, caused a significant reduction of the cell growth rate. The failure of compounds 3, 5, 8, 9, 21 and 25 to inhibit cell proliferation despite their potent inhibition of all three CDC25 phosphatases could be due to poor permeability into cells, chemical instability, unfavourable compartmentalization, active metabolism into inactive compounds, presence of unidentified binding proteins, or a combination of these factors. In conclusion, for its common behaviour in both melanoma cell lines, 7 was selected as the most promising compound for further investigation of its anti-melanoma effects.

CDC25s are fine regulators of the different phases of cell cycle. Hence, we checked if the reduction of cell growth rate caused by 7 was associated to an alteration of cell cycle progression. A significant reduction of the G0/G1 phase and an increase of the G2/M phase occurred in both melanoma cell lines after 16-h treatment and continued up to 24-h. It is known that many cytotoxic compounds, as well as various anti-cancer drugs, cause DNA damage [50–51]. This event can induce an arrest of cell proliferation, thus enabling cells to DNA repair in order to prevent cell death. On the other hand, if the mechanisms involved in the DNA repair are ineffective and the DNA damage is not completely repaired, cells can be committed to programmed cell death. As CDC25 proteins exert a pivotal role in the regulation of cell division cycle [8], their overexpression might facilitate checkpoint exit and contribute to neoplastic transformation. Hence, CDC25 inhibition, inactivation or degradation can amplify the cytotoxic activity of some compounds. The treatment of A2058 cells with 7 caused a modulation of the CDC25 protein levels. In particular, we observed an early and great reduction of CDC25A associated to a progressive decrease of CDC25C, that continued up to 16 h. These results suggest that the observed G2/M arrest of cell cycle in melanoma cells could depend on an inhibition of phosphatase activity of CDC25 proteins, as well as on a reduction of the protein levels of CDC25A and, more consistently, of CDC25C, as also observed in SAN cells. This behaviour seems in good agreement with the major role played by CDC25C in the control of the G2/M progression of cell cycle [52].

The reduction of cell growth rate and the G2/M block of cell cycle could evoke the beginning of a cell death program. Indeed, compound 7 activated a time-dependent apoptotic program in melanoma cells, as revealed by PI incorporation experiments and caspase-3 activity measurements. Hence, we can infer that the apoptosis observed in melanoma cells represents a consecutive process to the long cell cycle blockage in the G2/M phase.

It is known that quinonoid structures, as those of the most active CDC25 inhibitors, are good substrates for the beginning of a redox cycle, and that an increased ROS level may cause severe damages to the CDC25 structure [32, 53–54]. Indeed, quinonoid compounds exert their inhibitory activity on CDC25 through the oxidation of cysteine residues located in the active site [33, 36]. On the other hand, the increase of ROS level may be deleterious also to other cellular components, thus activating various metabolic pathways that control the cell cycle. Compound 7 increased the intracellular ROS levels in both melanoma cells, and this event was reverted by NAC, whereas apocynin, was ineffective. It is known that NAC is the precursor of glutathione, the main intracellular antioxidant, whereas the target of apocynin is NADPH oxidase, the most important ROS producer located on the membrane. On the basis of the different effect exerted by these two antioxidant molecules, we could speculate that the lipophilicity of 7 allows its efficient crossing through the cellular membrane, thus affecting the intracellular ROS level; however, the presence of some polar groups in 7 probably prevents its stable interaction with the inter-membrane components, such as NADPH oxidase. The finding that also the apoptosis was reduced by NAC suggests that the increase of intracellular ROS levels may be considered as an early event involved in the signalling triggered by compound 7.

The apoptotic machinery is an essential element of cell cycle checkpoints and the cytotoxic effects of many drugs are mediated in the mitochondrion through the activation of an intrinsic apoptotic pathway. Bcl-2 family members play a role not only in the regulation of apoptosis, but also in the control of cell cycle [55]. In this work, the observed increase of ROS levels, as well as the decrease of the crucial parameter for controlling life and death of a cell, *i.e*. the Bcl-2/Bax ratio, are clear markers of an involvement of the mitochondrion in the apoptotic program triggered by 7 in our cell systems. This hypothesis was further supported by the reduction of mitochondrial membrane potential, as well as by the increase of caspase-9 activity. Hence, our findings indicate that the alteration of the redox state induced by 7 could represent an underlying mechanism for the activation of the mitochondrial apoptotic pathway in melanoma cells.

Chemoresistance represents a typical hallmark of advanced melanomas. It has been reported that the aggressive nature of melanoma is related to an accumulation of mutations in several key proliferation- regulating mechanisms, as well as in apoptosis-controlling pathways [56]. Defects in Akt expression occur in a significant proportion of malignant melanomas [57]. Under this concern, the early reduction of the pAkt protein levels caused by 7 was very interesting, because it has been demonstrated that CDC25B mediates the activation of Akt, probably through a dephosphorylation mechanism of specific protein kinases [58–59]. A key molecule involved in the regulation of cell cycle and apoptosis pathway is p53. Frequently, in melanoma this protein is not mutated, but its impaired functions depend on high levels of the phosphorylated form of MDM2, a typical inhibitor of p53 [60]. The time-dependent decrease of the basal level of p53 observed in both melanoma cells, and its higher level measured in treated cells mainly after a late incubation with 7, suggest that this compound could affect the p53 protein stability. Indeed, higher levels of p53 in treated compared to untreated cells could be ascribable to a reduced activation of Akt, because this protein is responsible for the MDM2 phosphorylation [61–62]. On the other hand, it is likely that the reduced activation of Akt modulates the functions of other downstream proteins; under this concern, one possible candidate seems to be Bax, because of the increased levels of this pro-apoptotic factor observed upon treatment of A2058 with 7. In conclusion, we suggest that the early reduction of pAkt levels could be related to a concomitant impairment of CDC25 functions. In turn, the reduced activation of Akt could cause the deregulation of other downstream pathways leading to an increase of ROS level and later on of p53 levels.

Overall, our data indicate that the reduced viability of melanoma cells observed after treatment with compound 7 is probably related to the inhibition potency exhibited by this molecule on the CDC25 phosphatase activity, as well as to the modulation of its protein levels among the different forms. Therefore, the deregulation of CDC25 in melanoma cells suggests that this crucial element of cell cycle could be considered as a possible oncotarget *in vivo*. Under this concern, it is known that advanced anti-melanoma strategies are based on the usage of BRAF inhibitors, that selectively inhibit the proliferation of melanoma cells harbouring the BRAFV600E mutation [56]. However, the success of this therapy is not definitive, because usually the patients relapse because of acquired drug resistance, possibly due to the activation of others survival pathways. An alternative strategy could be represented by the combination of two different drugs, co-targeting independent survival pathways that are critical for development and maintenance of melanoma. Hence, the study of the effects of CDC25 inhibitors in melanoma cells could be helpful for finding other molecular pathways, as possible targets for melanoma treatment. The present work represents the fruitful combination of computational and biochemical work. The identification of a variety of inhibitors containing a 6-xanthone chemical motif for CDC25 phosphatase targets was made without the need for a massive high-throughput chemical screen. It is noteworthy that these tests were performed without the usage of robotics or highly automated methods, and the chemoinformatics and VS methods were performed on a common desktop computer. Thus, collaboration between (bio)chemical and VS provides an extraordinarily effective approach to drug discovery. A deeper insight in the molecular mechanisms of 7 in melanoma cells is under current investigation, in order to improve the structural and functional potency of this molecule; indeed, further refinement of this compound to higher affinity and more specific inhibition offers great therapeutic potential.

## MATERIALS AND METHODS

### Database: lead-like selection and preparation

The NCI Open Database (http://dtp.cancer.gov/) with 260.071 compounds was obtained from ZINC [39–40]. The compound database was processed with FILTER version 2.0.2 (OpenEye Scientific Software Inc., Santa Fe, USA, http://www.eyesopen.com/) to select a subset of lead-like compounds. We used the default parameters in the lead-like filter without further modifications. The resulting database, referred in this work as the NCI lead-like set, contained 65.375 compounds.

### Chemoinformatic methods

All of the approaches below were performed in parallel against the full ZINC drug-like subset (~17.8 million drug-like compounds) and the NCI lead-like set.

### Molecular fingerprints

Five types of molecular FPs ECFP2, ECFP4, FCFP2, FCFP4, and FCFP6 [63] were calculated using Pipeline Pilot (Accelrys Inc., San Diego, USA, http://accelrys.com/products/pipeline-Pilot/). Extended Connectivity Fingerprints (ECFPs) have been shown to have a number of strengths that make them useful for similarity searching. ECFPs are a FP methodology explicitly designed to capture molecular features relevant to molecular activity. They can be quickly calculated, as they are not defined a priori [63]. Functional Class Fingerprints (FCFPs) are a related fingerprint to ECFPs but instead of using a specific atom identifier for the initial atom in the algorithm to generate the fingerprint, FCFPs use a more abstract pharmacophoric set of initial atom identifiers based on properties such as H-bond acceptor (HBA) and donor (HBD), negatively and positively ionizable, aromatic, and halogen [63]. The similarity between the lead compound NSC 119915 and our compound libraries was assessed using the Tanimoto coefficient. The Tanimoto coefficient (*T_c_*) is given by eq. 1:

$$T_{c}(A\text{, }B)=\frac{c}{a+b-c}$$ (1)

where *a* and *b* are the number of bits set in the fingerprints of molecules *A* and *B*, respectively, and *c* is the number of bits set in both fingerprints. The *T_c_* ranges between 0 and 1, with 0 corresponding to no fingerprint overlap and 1 to identical fingerprints. It should be noted that, identical fingerprints do not necessarily correspond to identical molecules (as fingerprints are only abstractions of molecular structures). Furthermore, as defined by the above formula, the *T_c_* only takes into account bits set to 1 (i.e., features present in the molecule). Hence, the magnitude of the *T_c_* value will be greatly influenced by the bit density in the underlying fingerprint, which on the other hand, increases with molecular size and complexity [64]. The calculation of *T_c_* translates structural similarity into numerical values and can be interpreted as the “percentage of structural features shared between two compounds”, yet it is debatable which *T_c_* value corresponds to “significant similarity”. There is no generally applicable *T_c_* threshold for the indication of structural similarity, which is dependent on the molecular fingerprint applied [65]. In this work, we applied threshold values of 0.52, 0.43, 0.75, 0.60, 0.45 in combination with ECFP2, ECFP4, FCFP2, FCFP4, and FCFP6 fingerprints, respectively, because they give much higher confidence in correlating structural similarity [44].

### Substructure search

ZINC drug-like and NCI lead-like collections were exposed to substructure 2D searching using as query the following SMILES notations: [O = c3ccc2cc1ccccc1oc2c3], [O = c3ccc2cc1ccccc1[nH]c2c3] and [O = c3ccc2cc1ccccc1sc2c3]. Canvas version 1.9 (Schrödinger, LLC, New York, USA) was used in this process by using SMILES Arbitrary Target Specification (SMARTS) filter module to carry out substructure search.

All structures retrieved from both molecular fingerprints and substructure search were combined and duplicates were removed, thus obtaining a single database of 126 unique compounds. Out of these compounds, we selected the top-ranked 25 compounds for CDC25 inhibitory assay.

### Materials and reagents

All compounds were purchased from commercial vendors or kindly provided from the NCI/DTP. Compounds were dissolved in DMSO, and stock solutions at 10 mM concentration were prepared. Recombinant forms of the catalytic domains of CDC25A, -B and -C were obtained through the vectors pET28a-CDC25A-cd, pET28a-CDC25B-cd and pET28a-CDC25C-cd, kindly provided by H. Bhattacharjee (Florida International University, Herbert Wertheim College of Medicine, Miami, Florida). Protein purification was obtained essentially as previously described [36]. The synthetic substrate for CDC25 phosphatase activity, OMFP, was purchased from Sigma-Aldrich. Dulbecco's modified Eagle's medium (DMEM), Roswell Park Memorial Institute (RPMI) 1640 medium, fetal bovine serum (FBS), L-glutamine, penicillin G, streptomycin, and trypsin were purchased from Lonza (Milano, Italy). Propidium iodide (PI), dichlorofluorescein diacetate (DCFH-DA), Rhodamine 123 (R123), N-acetyl-L-cysteine (NAC) and apocynin were purchased from Sigma-Aldrich. A protease inhibitor cocktail was obtained from Roche Diagnostics S.p.A. (Monza, Italy). Caspase-3 and caspase-9 fluorimetric assay kits were purchased from BioVision (Milpitas, CA, USA). The pan-caspase inhibitor Z-VAD-FMK was purchased from Selleckchem (USA). Rabbit monoclonal antibody against glyceraldehyde 3-phosphate dehydrogenase (GAPDH) was obtained from Cell Signaling (Boston, MA, USA); mouse monoclonal antibody against CDC25A, CDC25C, or Bcl-2, rabbit polyclonal antibody against CDC25B, pAkt (Ser473) or Bax, and each secondary antibody conjugated to horseradish peroxidase were obtained from Santa Cruz Biotechnology (Heidelberg, Germany). All other chemicals were of analytical grade and were purchased from Sigma-Aldrich.

### *In vitro* assays of CDC25 phosphatase activity

The enzymatic activity of the catalytic domains of CDC25A, -B and -C were determined through a fluorimetric method, which monitored the dephosphorylation of the synthetic substrate OMFP, essentially as previously described [36]. In steady-state enzyme kinetic studies, the residual phosphatase activity of purified recombinant CDC25B was measured at 30°C in the presence of different concentrations of the various inhibitors, using a computer-assisted Cary Eclipse spectrofluorimeter (Varian) equipped with an electronic temperature controller. Excitation and emission wavelengths were set at 485 and 530 nm, respectively; both excitation and emission slits were set at 10 nm. The reaction mixture contained 10 nM CDC25B and different concentrations of the various inhibitors in 500 μL final volume of 20 mM Tris-HCl, pH 7.8, 1 mM DTT. DMSO was used as vehicle control. The reaction started by the addition of 25 μM OMFP, and the formation of the fluorescent product *o*-methylfluorescein was monitored continuously. The rate of OMFP hydrolysis was expressed as arbitrary units per min (a.u./min). The comparison of the rates determined in the absence and in the presence of the various inhibitors allowed the calculation of the residual phosphatase activity, expressed as a percentage.

To measure the inhibition constant (*K*_i_) of the recombinant forms of CDC25A, -B and -C towards the various inhibitors, the affinity of the different forms of CDC25 towards OMFP was measured either in the absence or in the presence of fixed concentrations of the various inhibitors. The reaction mixture contained 20 nM CDC25A, or 10 nM CDC25B, or 40 nM CDC25C, and different concentrations of the various inhibitors in 500 μL final volume of 20 mM Tris-HCl, pH 7.8, 1 mM DTT. DMSO was used as vehicle control. The reaction started by the addition of 1–25 μM OMFP, and the rate of OMFP hydrolysis was measured as indicated before. The corresponding Lineweaver-Burk plots allowed the calculation of the *K*_M_ for OMFP and of the *V*_max_ of OMFP hydrolysis, expressed as a.u./min_max_. In the presence of the various inhibitors the *K*_M_ value for OMFP remained essentially unchanged, whereas the *V*_max_ decreased, thus indicating that the selected compounds were noncompetitive inhibitors of CDC25. The *K*_i_ values were obtained from the decrease of a.u./min_max_ in the presence of the inhibitor, according to the equation a.u./mim_max_’ = a.u./mim_max_/{1 + ([I]/K_i_)}, where a.u./mim_max_’ represents the *V*_max_ measured in the presence of the concentration [I] of the inhibitor. The *K*_i_ values were obtained from at least three independent experiments and reported as mean ± S.E.

### Cell culture

The human melanoma cell line A2058, kindly provided by CEINGE (Naples, Italy), and SAN cells [66] were derived from lymph nodal metastases and grown in DMEM and RPMI 1640, respectively, supplemented with 10% FBS, 2 mM L-glutamine, 100 IU/mL penicillin G, and 100 μg/mL streptomycin in humidified incubator at 37°C under 5% CO_2_ atmosphere. All cells were split and seeded every three days and used during the exponential phase of growth. Cell treatments were always carried after 24 h from plating.

### 3-(4,5-Dimethylthiazole-2-yl)-2,5-biphenyltetrazolium bromide (MTT) assay

The MTT assay was used to detect cell proliferation essentially as previously described [67]. Briefly, A2058 and SAN cells were plated in 96-well microtiter plates (100 μL/well) at 4000 and 6000 cells/well, respectively. After 24-h seeding, cells were treated with the selected compounds added at 25, 50 or 100 μM concentration, or with 0.5% (v/v) DMSO as a vehicle control. After 24-h, 48-h or 72-h treatment, and upon the addition of 10 μL of MTT solution in the dark, the plate was incubated for 3 h at 37°C under CO_2_ atmosphere. After medium aspiration and solubilization of formazan crystals, absorbance was measured at 570 nm, using an ELISA plate reader (Bio-Rad, Milano, Italy).

### Cell cycle analysis and evaluation of apoptosis

Cells were seeded into 6-well plates at 3 × 10^5^ cells/well for 24 h at 37°C; after the addition of 100 μM 7 or 0.5% DMSO as a vehicle control, the incubation of treated cells continued for 16 or 24 h. After each treatment, cells were harvested with trypsin, centrifuged and the pellet was resuspended in phosphate-buffered saline (PBS). For cell cycle analysis, cells were fixed with 70% (v/v) cold ethanol and stored at −20°C for 1 h. Then, cells were washed with cold PBS, centrifuged and the pellets were resuspended in 200 μL of a non-lysis solution containing 50 μg/mL PI. For the evaluation of apoptosis, cells were not fixed in ethanol and directly resuspended in 200 μL of a hypotonic lysis solution containing 50 μg/mL PI. After incubation at 4°C for 30 min, cells were analyzed with a FACScan flow cytometer (Becton Dickinson) for evaluating the distribution in cell cycle phases or the presence of nuclei with a DNA content lower than the diploid.

### Measurements of caspase-3 and caspase-9 activity

To estimate caspase-3 and caspase-9 activity during the treatment with compound 7, the respective enzymatic activities were measured by using caspase-3 and -9 fluorimetric assay kits, according to the manufacturer's protocol, essentially as previously described [68]. Briefly, cells were seeded into 75 cm^2^ plates (2 × 10^6^ cells/plate) for 24 h at 37°C and then treated with 100 μM 7 or 0.5% DMSO. At the end of each incubation, cells were collected, washed with PBS, and finally lysed at 4°C in the cell lysis buffer. Cell lysates were incubated with 50 μM DEVD-AFC or LEHD-AFC substrates at 37°C for 2 h, to detect caspase-3 or caspase-9 activity, respectively, using a Cary Eclipse fluorescence spectrophotometer (Varian). Excitation and emission wavelengths were set at 400 nm and 505 nm, respectively; both excitation and emission slits were set at 10 nm.

### Measurement of intracellular ROS content

The intracellular ROS level was monitored using the oxidation-sensitive fluorescence probe DCFH-DA. Cells were seeded into 6-well-plates (3 × 10^5^ cells/plate) for 24 h at 37°C and then treated at various times with 100 μM compound 7 or 0.5% DMSO. DCFH-DA was added in the dark at 10 μM final concentration 30-min before the end of each incubation; then, cells were collected, washed in PBS, and finally resuspended in 500 μL PBS for fluorimetric analysis. Measurements were realized in a Cary Eclipse fluorescence spectrophotometer (Varian); excitation and emission wavelengths were set at 485 nm and 530 nm, respectively; both excitation and emission slits were set at 10 nm. The effect of 7 on ROS production was also estimated after pretreatment of cells with 10 mM NAC for 1 h.

### Evaluation of mitochondrial membrane potential

Mitochondrial membrane potential was evaluated by measuring the incorporation of the fluorescent probe R123, essentially as previously described [69]. Briefly, cells were seeded into 6-well-plates (3 × 10^5^ cells/well) for 24 h at 37°C, and then incubated at 37°C for 1 h in the presence of 5 μM R123, washed twice with PBS, and placed in medium containing 100 μM compound 7 or 0.5% DMSO. After various times from treatment, cells were harvested, washed and centrifuged for 10 min at 4°C. The cellular pellet was resuspended in 500 μL PBS. The fluorescence of cell-associated R123 was detected in the above-mentioned fluorescence spectrophotometer, using excitation and emission wavelengths of 490 and 520 nm, respectively; both excitation and emission slits were set at 10 nm.

### Western blotting

A2058 cells were seeded into 6-well-plates (3 × 10^5^ cells/plate) for 24 h at 37°C and then treated at different times with 100 μM compound 7 or 0.5% DMSO. After treatment, cells were harvested, washed with PBS and then lysed in ice-cold modified radio immunoprecipitation assay (RIPA) buffer (50 mM Tris-HCl, pH 7.4, 150 mM NaCl, 1% Nonidet P-40, 0.25% sodium deoxycholate, 1 mM Na_3_VO_4_ and 1 mM NaF), supplemented with protease inhibitors and incubated for 30 min on ice. The supernatant obtained after centrifugation at 12,000 rpm for 30 min at 4°C constituted the total protein extract. The protein concentration was determined by the method of Bradford, using bovine serum albumin (BSA) as standard [70]. Equal amounts of total protein extracts were used for Western blot analysis. Briefly, protein samples were dissolved in SDS-reducing loading buffer, run on 12% SDS/PAGE and then transferred to Immobilon P membrane (Millipore). The filter was incubated with the specific primary antibody at 4°C overnight and then with the secondary antibody at room temperature for 1 h. Membranes were then analysed by an enhanced chemiluminescence reaction, using Super Signal West Pico kit (Pierce) according to manufacturer's instruction; signals were visualized by autoradiography.

### Statistical analysis

Data are reported as average and standard error. The statistical significance of differences among groups was evaluated using ANOVA, with the Bonferroni correction as post hoc test or the Student *t* test where appropriate. The significance was accepted at the level of *p* < 0.05.

## SUPPLEMENTARY FIGURES AND TABLES



## Abbreviations

Akt: protein kinase B
Bax: Bcl-2-associated X protein
Bcl-2: B-cell lymphoma-2
BSA: bovine serum albumin
CDC25: cell division cycle 25
CDK: cyclin-dependent kinases
DCFH-DA: dichlorofluorescein diacetate
DMEM: Dulbecco's modification of Eagle's medium
DMSO: dimethyl sulfoxide
DSP: dual-specificity phosphatase
DTT: dithiothreitol
DTP: Developmental Therapeutics Program
ECFP: Extended Connectivity Fingerprint
FBS: foetal bovine serum
FCFP: Functional Class Fingerprint
GAPDH: glyceraldehyde 3-phosphate dehydrogenase
MDM2: human murine double-minute 2 protein
MTT: 3-(4,5-Dimethylthiazole-2-yl)-2,5-biphenyltetrazolium bromide
NAC: N-acetyl-cysteine
NCI: National Cancer Institute
OMFP: 3-O-methylfluorescein phosphate
PBS: phosphate-buffered saline
PI: propidium iodide
RIPA: radio immunoprecipitation assay
R123: Rhodamine 123
ROS: reactive oxygen species
SAR: structure-activity relationship
SDS/PAGE: sodium dodecyl sulfate polyacrylamide gel electrophoresis
SMARTS: SMILES Arbitrary Target Specification
VS: virtual
