# Supplementary material for: Ligand-based chemoinformatic discovery of a novel small molecule inhibitor targeting CDC25 dual specificity phosphatases and displaying in vitro efficacy against melanoma cells
Source: Oncotarget. 2015 Oct 13;6(37):40202–22. doi: 10.18632/oncotarget.5473 (PMC4741889; doi:10.18632/oncotarget.5473)
Supplement: Supplementary file 1 [file oncotarget-06-40202-s001.pdf]

SUPPLEMENTARY FIGURES

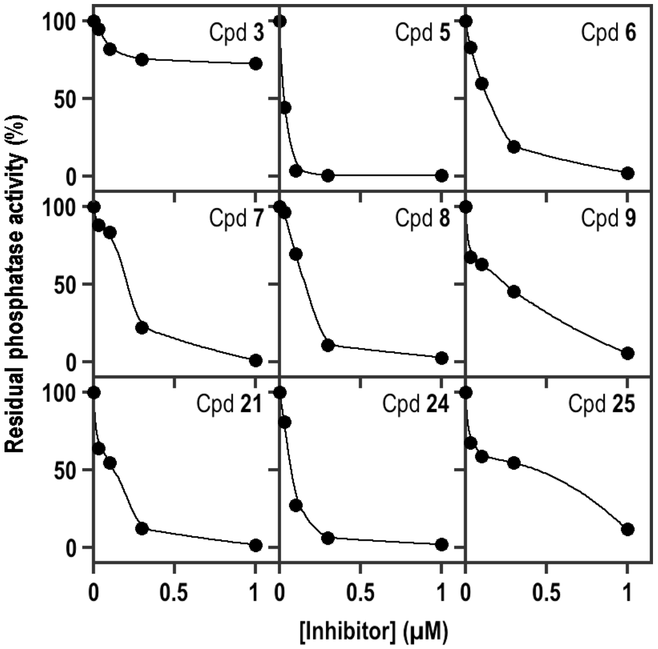

Supplementary Figure S1: Dose-dependent inhibition profile by the selected CDC25B inhibitors. The phosphatase activity of 10 nM CDC25B was measured through the rate of OMFP hydrolysis in the presence of 25 μM substrate and the indicated concentration of the compounds 3, 5–9, 21, 24–25. The activity was expressed as a percentage of that measured in the absence of inhibitor. Other details are described in the Materials and Methods.

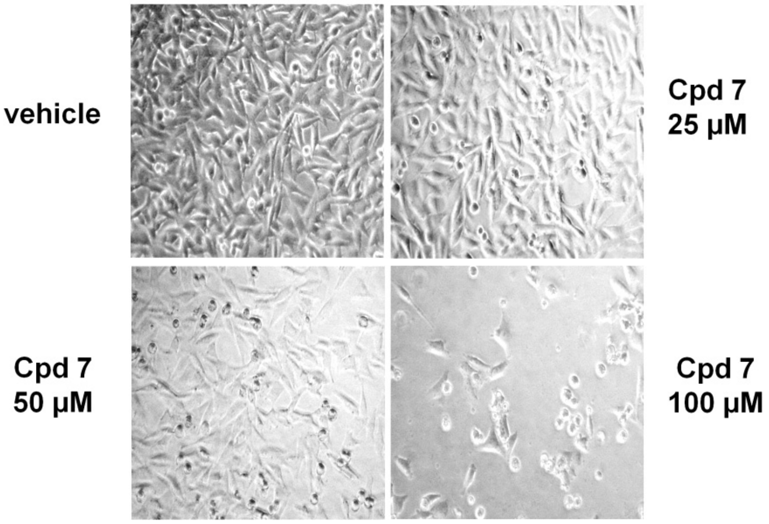

Supplementary Figure S2: Morphological changes of A2058 cells induced by compound 7. Cells were incubated for 48 h with vehicle alone or 25, 50, 100 μM compound 7. Images are representative of three independent experiments. Magnification × 10.

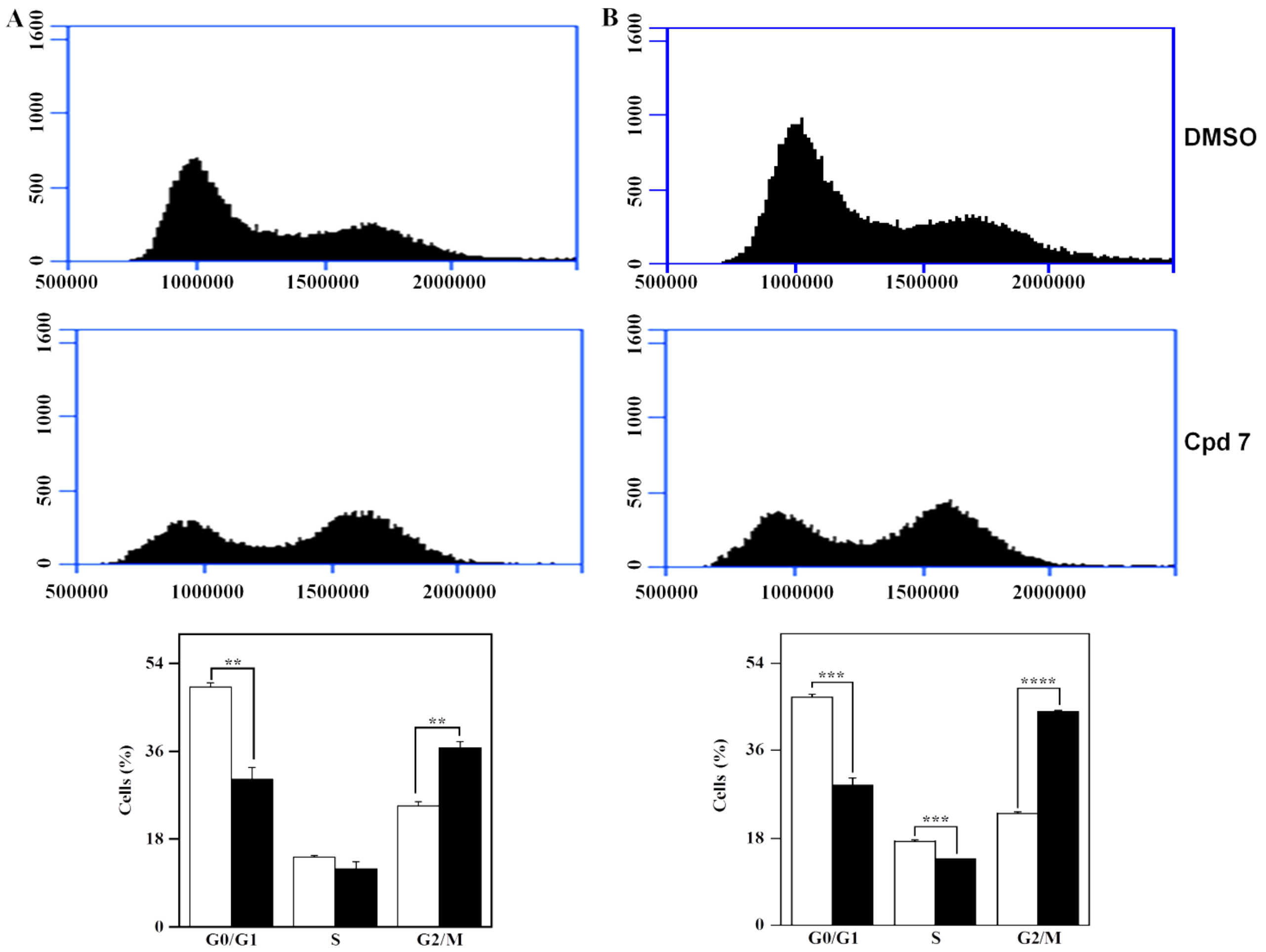

Supplementary Figure S3: Effect of compound 7 on the distribution of cell cycle phases of SAN cells. The determination of cells in the different phases was evaluated after **A.** 16 h or **B.** 24 h from treatment with 0.5% DMSO or 100 µM compound 7, as described in the Materials and Methods. Histograms, which show the cell percentage among the various phases, were obtained from triplicate experiments and reported as the means ± SE. $^{**}p < 0.01$, $^{***}p < 0.001$ and $^{****}p < 0.001$ compared to control cells. Vehicle alone, open bars; compound 7, black bars.

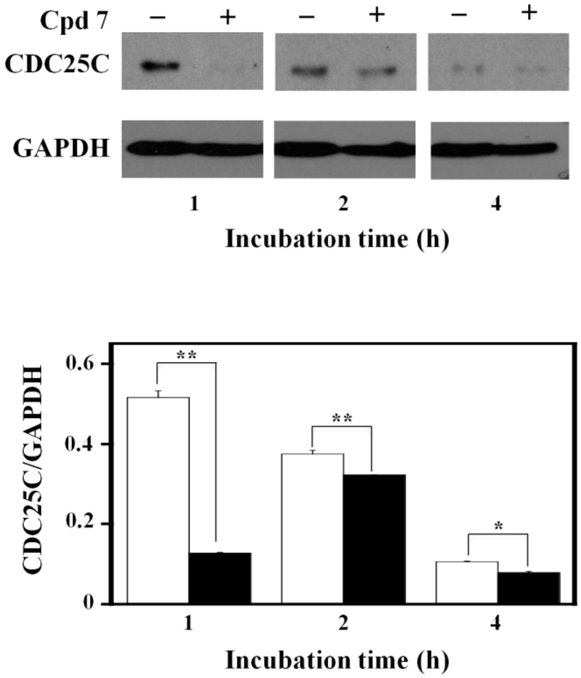

Supplementary Figure S4: Effect of compound 7 on CDC25C protein levels. Total protein extracts from SAN cells, incubated with 0.5% DMSO (open bars) or 100 μM compound 7 (black bars) for 1, 2, or 4 h, were analyzed by Western blotting. GAPDH was used as loading control. Densitometric analysis is shown in the lower panel. Data from triplicate experiments were reported as the means ± SE. *$p < 0.05$, **$p < 0.01$, compared to control cells. Other details as described in the Materials and Methods.

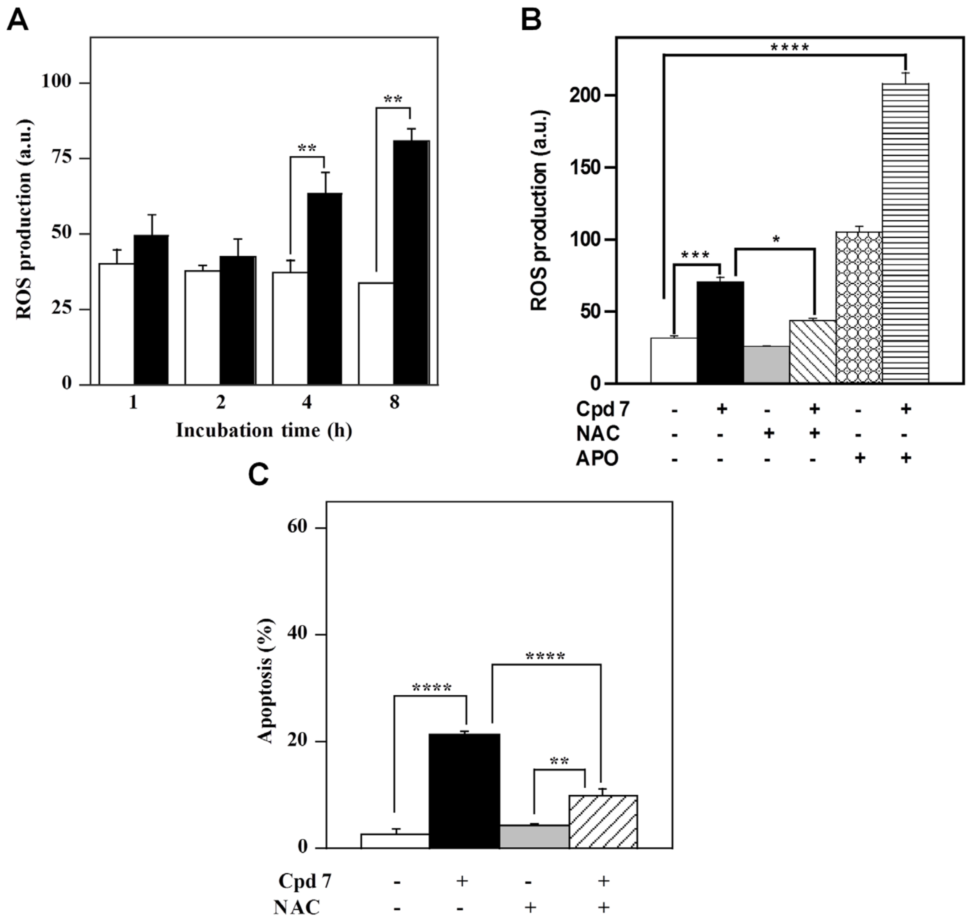

Supplementary Figure S5: ROS production and their involvement in the apoptotic process of SAN cells, as induced by treatment with compound 7. A. Time-dependent measurement of ROS production. Cells were incubated with 0.5% DMSO (open bars) or 100 µM compound 7 (black bars) and then the intracellular ROS level was measured. **B.** Effect of antioxidant molecules on ROS production. The ROS level was also measured in cells untreated or pretreated with NAC or apocynin after a 4-h incubation with DMSO or 7. **C.** Effect of NAC on apoptosis. The PI incorporation was evaluated in cells untreated or pretreated with NAC after a 48-h incubation with DMSO or 7. ROS production was expressed as a.u., and apoptosis as a percentage. Data from triplicate experiments were reported as the means ± SE. **p* < 0.05, ***p* < 0.01, ****p* < 0.001 and *****p* < 0.0001 compared to relative control cells. Other details as described in the Materials and Methods.

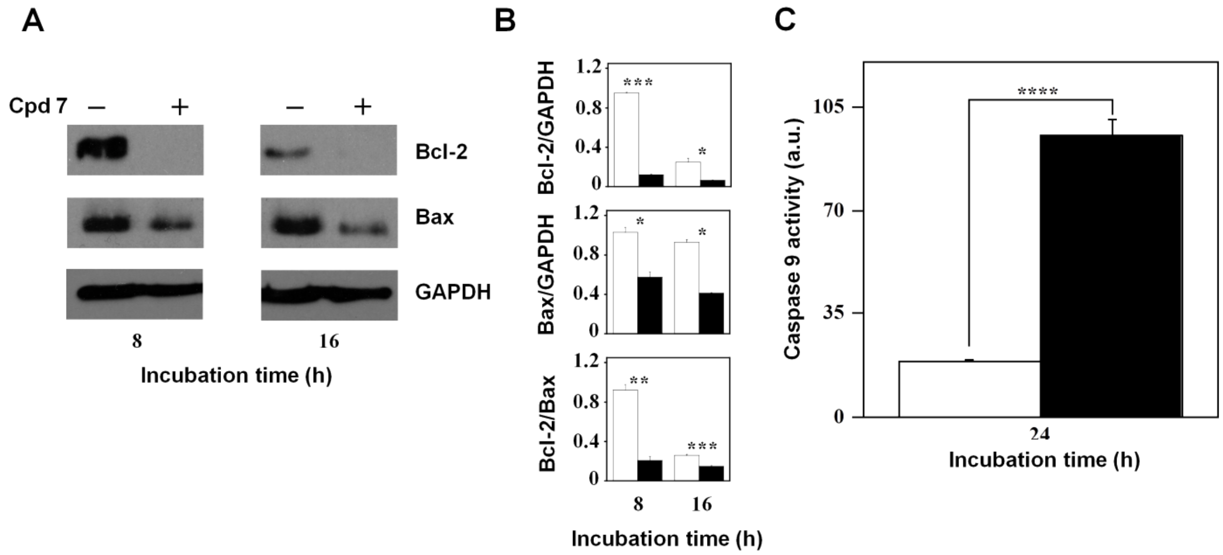

Supplementary Figure S6: Effect of compound 7 on some apoptotic mitochondrial markers in SAN cells. A. Evaluation of Bcl-2 and Bax protein levels. Total protein extracts from SAN cells, incubated with 0.5% DMSO or 100 µM compound 7 for 8 or 16 h,were analyzed by Western blotting. GAPDH was used as loading control. **B.** Densitometric analysis of the Bcl-2 and Bax protein levels, as well as of the Bcl-2/Bax ratio. **C.** Determination of the caspase-9 enzymatic activity. Total protein extracts from SAN cells, incubated with 0.5% DMSO (open bars) or 100 µM compound 7 (black bars) for 24 h, were assayed for caspase-9 activity. Data from triplicate experiments were reported as the means ± SE. *$p < 0.05$, **$p < 0.01$, ***$p < 0.001$,****$p < 0.0001$, compared to control cells. Other details as described in the Materials and Methods.

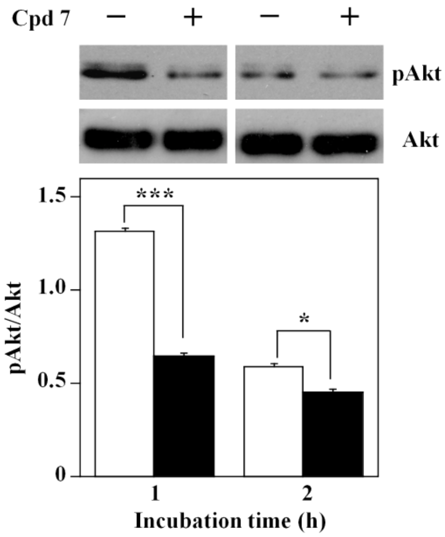

Supplementary Figure S7: Effect of compound 7 on pAkt protein levels. Total protein extracts from SAN cells, incubated with 0.5% DMSO or 100 μM compound 7 for 1 or 2 h, were analyzed by Western blotting using an antibody raised against pAkt (Ser473). Akt was used as loading control. Densitometric analysis shown in the lower panel. Data from triplicate experiments were reported as the means ± SE. *p < 0.05, ***p < 0.001, compared to control cells. Other details as described in the Materials and Methods.
